# Giant Pheochromocytoma Resection Using Partial Cardiopulmonary Bypass and Blood Purification Therapy

**DOI:** 10.1210/jcemcr/luae202

**Published:** 2024-10-24

**Authors:** Hiroyuki Otsuka, Eiki Tayama, Keiichiro Uemura, Hiro Furusaka, Toru Hisaka, Tsukasa Igawa

**Affiliations:** Department of Surgery, Division of Cardiovascular Surgery, Kurume University School of Medicine, Kurume, Fukuoka, Japan 830-0011; Department of Surgery, Division of Cardiovascular Surgery, Kurume University School of Medicine, Kurume, Fukuoka, Japan 830-0011; Department of Urology, Kurume University School of Medicine, Kurume, Fukuoka, Japan 830-0011; Department of Urology, Kurume University School of Medicine, Kurume, Fukuoka, Japan 830-0011; Department of Surgery, Division of Hepatobiliary Pancreas Surgery, Kurume University School of Medicine, Kurume, Fukuoka, Japan 830-0011; Department of Urology, Kurume University School of Medicine, Kurume, Fukuoka, Japan 830-0011

**Keywords:** giant pheochromocytoma, partial CPB, blood purification therapy

## Abstract

A 44-year-old man was diagnosed with a giant pheochromocytoma in the right retroperitoneal cavity following treatment for heart failure. Subsequent to improvement in cardiac function, the patient underwent a laparotomy to excise the tumor. Due to its considerable size, partial cardiopulmonary bypass and blood purification therapy were initiated to stabilize hemodynamics during the surgical intervention. Herein, we present the utilization of partial cardiopulmonary bypass and blood purification in the resection of a giant pheochromocytoma in a patient with a history of heart failure, which proved beneficial in ensuring hemodynamic stability.

## Introduction

Pheochromocytoma/paraganglioma (PPGL) is a rare yet profoundly substantial tumor originating from chromaffin cells within the adrenal medulla or extra-adrenal paraganglia. These tumors can induce changes in myocardial structure and function, ultimately leading to the onset of severe cardiomyopathy [1]. Presently, surgery remains the preferred course of treatment [2]. PPGL is characterized by heightened hemodynamic instability and increased cardiovascular mortality due to catecholamine release [3]. Particularly, these risks are amplified during surgical procedures, especially during anesthetic induction and excision of large tumors, where the massive release of catecholamine can trigger hypertensive crises. Partial cardiopulmonary bypass (CPB) is a medical technique that temporarily takes over part of the cardiac function, specifically maintaining circulation and oxygenation. By using an external machine, it helps stabilize blood flow and pressure during surgeries where these factors may become unstable. Blood purification therapy is a treatment that eliminates waste products and excess fluids from the blood, effectively removing catecholamines (adrenaline and noradrenaline) released by tumors, especially during surgeries for pheochromocytoma.

Herein, we present the successful execution of surgery for a giant pheochromocytoma complicated by heart failure, accomplished through the strategic utilization of partial CPB and blood purification therapy.

## Case Presentation

A 44-year-old man presented at our hospital for management of heart failure and a retroperitoneal tumor. He had been experiencing intermittent palpitations and dizziness with increasing frequency over the past 2 months, prompting him to seek consultation with his primary care physician. The patient had no family history of malignancies or hereditary syndromes. On admission to the primary care hospital, his systolic blood pressure was 60 mm Hg, and his oxygen saturation level was low (sPO_2_: 80% on room air). He was promptly started on oxygen therapy and antibiotics and administered 60 mg of intravenous prednisolone. Electrocardiography revealed a normal sinus rhythm, showing no findings suggestive of acute coronary syndrome. Chest radiography showed a cardiothoracic ratio of 54% (normal range, <50%) with signs of pulmonary congestion, and echocardiography indicated a left ventricular ejection fraction (LVEF) of 30%, suggestive of severe heart failure. Thoracoabdominal computed tomography revealed a large right-sided retroperitoneal tumor. Following acute treatment and stabilization of his condition, he was referred to our hospital for further management of heart failure and the retroperitoneal tumor.

## Diagnostic Assessment

**Figure 1.**
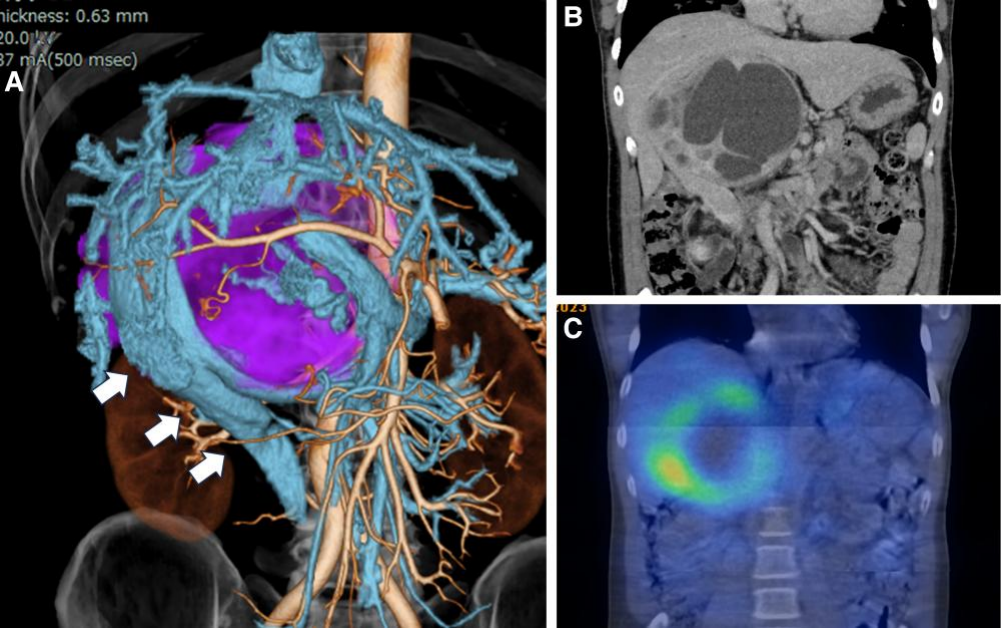
Abdominal 3-dimensional computed tomography revealing a right adrenal mass measuring 16 × 11 × 12 cm^3^ draining into the inferior vena cava, indicated by A, the white arrow. B, The tumor presents as a giant right pheochromocytoma with central necrosis. C, Scintigraphy scan with ^123^I-metaiodobenzylguanidine demonstrates uptake mainly at the tumor margins.

Laboratory investigations revealed elevated plasma levels of free normetanephrine at 3960 pg/mL (21 621.6 pmol/L) (normal reference, < 506.0 pg/mL; < 2762.76 pmol/L) and free metanephrine at 2080pg/mL (10 545.6 pmol/L) (normal reference, < 130.0 pg/mL; < 659.1 pmol/L). Additionally, urinary metanephrine levels were elevated at 21 240 μg/day (107 686.8 nmol/d) (normal reference, 40-190 μg/day), and urinary normetanephrine levels were elevated at 9230 μg/day (50 395.8 nmol/d) (normal reference, 90-330 μg/day). Abdominal computed tomography revealed a septated cystic tumor measuring approximately 16 × 11 × 12 cm^3^, compressing the duodenum and the inferior vena cava from the dorsal side in the right retroperitoneal cavity. Scintigraphy scan with ^123^I-metaiodobenzylguanidine (Fig. 1) and ^18^F-fluorodeoxyglucose positron emission tomography showed uptake mainly within the peripheral solid tumor component, with no evidence of metastatic lesions. Based on the aforementioned biochemical and radiological findings, the patient was diagnosed with pheochromocytoma.

## Treatment

After admission, he experienced recurrent episodes of hypotension and paroxysmal hypertension. Consequently, doxazosin (2 mg/day) and metyrosine (starting dose of 1000 mg/day) were administered, with subsequent adjustment of the daily dose of metyrosine up to a maximum of 3000 mg/day. However, the doxazosin dose could not be up titrated due to wide fluctuations in blood pressure and severe heart failure. In instances of uncontrolled hypertension, the patient received treatment via continuous intravenous infusion of phentolamine (0.125 mg/mL). Surgery was then scheduled after his cardiac function was restored (LVEF: 73%). A multidisciplinary team comprising specialists in urology, hepatobiliary and pancreatic surgery, cardiovascular surgery, anesthesiology, and endocrinology deliberated on the surgical approach. Ultimately, a consensus was reached to proceed with open tumor resection using partial CPB and blood purification therapy.

**Figure 2.**
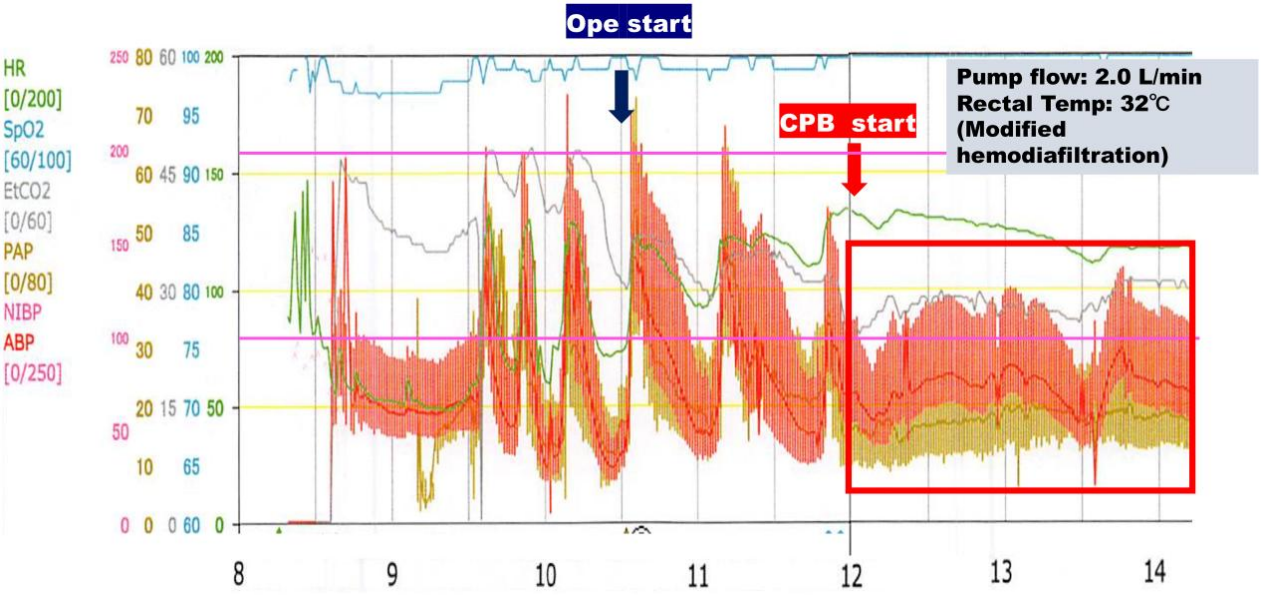
Intraoperative blood pressure course during partial cardiopulmonary bypass (CPB).

**Figure 3.**
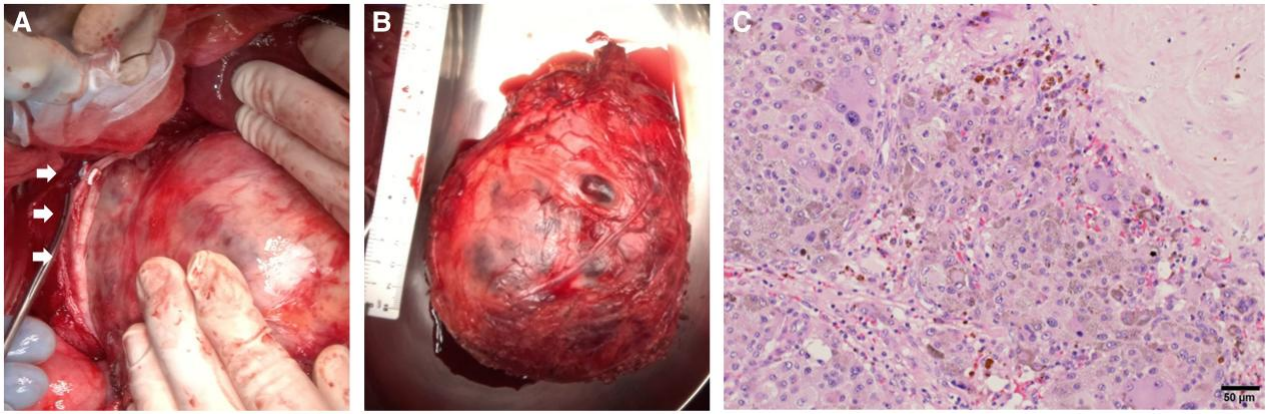
An intraoperative finding of the tumor draining into the inferior vena cava, denoted by A, the white arrow. B, An excised sample. Light micrograph of a hematoxylin-and eosin-stained section. C, The tumor cells were composed of large polygonal cells, and formed a wide cytoplasm that was granular in appearance and showed bilateral affinity.

On entering the operating room, general anesthesia was initiated; however, the patient exhibited considerable variability in blood pressure, fluctuating between 60 and 200 mm Hg (Fig. 2). Prior to surgery, an oblique incision was made in the right inguinal region to access and secure the right femoral artery and vein for cannulation of the partial CPB circuit. Afterward, the abdomen was opened via a reverse-L incision from the midline of the upper abdomen to the posterior right costal arch. Mobilization of the right lobe of the liver then ensued, followed by retraction of the ascending colon and incision of the retroperitoneum. Subsequently, the hepatic mesentery was incised, and the space between the anterior lobe of the Gerota fascia and the mesentery of the colon was dissected. Throughout the surgical procedure, efforts were made to manage episodes of severe hypertension (systolic blood pressure ≥ 180 mm Hg), primarily using α-receptor blockers; however, hemodynamics remained highly unstable. Consequently, partial CPB was initiated before peritumoral manipulation, following full heparinization of the right femoral artery and vein. Under fluoroscopic guidance, a venous catheter was inserted to a depth of 18 cm and positioned at the lower end of the tumor. Blood purification therapy was performed continuously via the venous catheter until the end of surgery. With partial CPB initiated, a flow rate of 2 L/min was maintained with mild hypothermia (bladder temperature of 32 °C), ensuring stable blood pressure (∼110 mm Hg) throughout the procedure via volume adjustment (see Fig. 2). The tumor was carefully dissected from the surrounding tissues employing a vessel-sealing system (LigaSure) before being excised (Fig. 3). Notably, despite the relatively aggressive manipulation of the tumor, hemodynamics remained stable. Following tumor removal, partial CPB was smoothly discontinued, and given the separation of the left renal vein from the inferior vena cava during tumor resection, reconstruction of the right renal vein and inferior vena cava separation site was achieved with a 5-0 Prolene suture via an end-to-side anastomosis. Likewise, hemostasis was easily achieved following heparin reversal using protamine. The excised tumor weighed 860 g. However, this weight was lighter than the actual weight due to the intraoperative rupture of the capsule (operation time: 7 hours 28 minutes; partial CPB time: 4 hours and 6 minutes). Microscopic examination of hematoxylin and eosin–stained sections showed that the tumor cells were composed of large polygonal cells and formed a wide cytoplasm that was granular in appearance and showed bilateral affinity (see Fig. 3).

## Outcome and Follow-up

Postoperatively, the patient maintained stable hemodynamics and was successfully extubated on postoperative day 1. Although low-dose noradrenaline (0.14μg/kg/min) was administered until the following day, vasopressor/ionotropic support was not required thereafter. Oral intake commenced on postoperative day 3; however, due to chyle drainage from the intraperitoneal drain, the patient fasted for 3 weeks and recovered with conservative care. The patient's recovery progressed favorably, leading to discharge from the hospital on postoperative day 39. Subsequent outpatient follow-ups were scheduled with the urology and endocrinology departments. At the 6-month postoperative mark, the patient remained in good health, with no signs of recurrence.

## Discussion

PPGLs are rare neuroendocrine tumors that produce excessive catecholamines and originate from the adrenal gland or extra-adrenal chromaffin cells in the sympathetic and parasympathetic ganglia [4]. Excessive catecholamines can induce severe vasoconstriction, coronary vasospasm, myocardial ischemia, injury, and necrosis, leading to various clinical manifestations. Additionally, excessive catecholamines may cause damage to cardiac myocytes, resulting in conditions such as takotsubo cardiomyopathy, inverted takotsubo cardiomyopathy, hypertrophic cardiomyopathy, myocarditis, and dilated cardiomyopathy [4, 5]. While catecholamine-induced cardiomyopathy is relatively rare, affecting 8% to 11% of patients with pheochromocytoma [6], it poses considerable challenges in management [1, 4, 5, 7]. The mechanisms underlying myocyte damage involve coronary dysfunction associated with α-receptor activation [8], downregulation of β-receptors [9], increased blood coagulation and platelet aggregation [10], intramyocardial calcium overload [11], and the secretion of various factors by pheochromocytoma tumor cells [12]. In the present case, following the exclusion of coronary artery syndrome, based on the presence of a retroperitoneal tumor and elevated urinary metanephrine and normetanephrine levels, we established a definitive diagnosis of catecholamine-induced cardiomyopathy secondary to pheochromocytomas. The management of catecholamine-induced cardiomyopathy involves stabilizing blood pressure with α-adrenoceptor blockers, followed by the addition of β-adrenoceptor blockers, and ultimately, surgical resection of the pheochromocytoma once the patient is clinically stable [13]. Central to therapeutic management is the use of α-adrenoceptor blockade to inhibit the effects of catecholamines on α-adrenoceptors, thereby regulating various organ functions. To prevent unopposed α-adrenergic action, β-adrenoceptor blockers are introduced to manage tachycardia, but only after appropriate α-adrenoceptor blockade [2]. Calcium blockers and nitrates can also be employed for hypertension management, with the addition of β-blockers if tachycardia occurs. Metyrosine treatment has shown remarkable efficacy in reducing catecholamines in patients with PPGL, and adjusting its administration can enhance the effectiveness of surgical pretreatment by stabilizing blood pressure and improving glucose metabolism [14]. Additionally, treatment may include a high-sodium diet and increased fluid intake to counteract catecholamine-induced blood volume contractions preoperatively and prevent severe hypotension after tumor excision [2]. Preoperative optimization with α-blockers, the first-line treatment for PPGL, was not feasible in this case due to marked blood pressure fluctuations and severe heart failure. Ideally, surgical resection of the PPGL should be performed after stabilization of the patient's general condition following these medical interventions within 7 to 14 days [2]. However, despite the use of life-support devices, some pheochromocytoma crises can be fatal. Notably, in the present case, metyrosine addition served as a valuable adjunct in preoperative management when escalating α-blocker doses proved difficult. Consequently, while minimally invasive adrenalectomy (eg, laparoscopic) is suitable for most pheochromocytomas, open adrenalectomy is recommended in specific situations, such as tumors larger than 6 cm, those with central necrosis (prone to rupture), or those located near major blood vessels [15].

Generally, extracorporeal circulation is rarely required for pheochromocytoma removal. However, in this case, the tumor was exceptionally large and exhibited necrotic features, raising concerns that catecholamines could easily enter the bloodstream during surgical manipulation for removal, causing abnormal hemodynamics. To mitigate this risk, partial CPB was employed to regulate blood pressure, manage blood volume, and facilitate blood purification therapy simultaneously. The utility of CPB in the surgical removal of pheochromocytomas involving the heart is well established [16, 17]; however, its application in pheochromocytoma removal remains rare. Surgical challenges arise from the need for extremely careful manipulation, as minimal contact with the tumor can trigger hormone release. Partial CPB offers the advantage of substantially attenuating blood pressure fluctuations induced by tumor manipulation [16]. In the present case, volume management with partial CPB effectively stabilized hemodynamics. Furthermore, in partial CPB with full heparinization, bleeding can be collected and reused intraoperatively, aiding in intraoperative bleeding management. Thus, partial CPB is a beneficial means of preventing acute hemodynamic fluctuations caused by catecholamine release and massive bleeding due to surgical maneuvers, eliminating the need for α-blocker administration and improving surgical accuracy and safety. Moreover, controlling central venous pressure at low levels minimized venous bleeding during dissection, and subsequent protamine administration post partial CPB ensured hemostasis without complications.

Additionally, continuous hemodiafiltration, a recognized treatment for catecholamine crises [18] [19], was employed in this case to address the abnormally high catecholamine levels observed during surgery. Interestingly, intraoperative catecholamine levels showed an increase with tumor manipulation and a gradual decrease after tumor explant ((Table 1 and Fig. 2).

**Table 1.** Intraoperative blood catecholamine levels in our patient

Hormone tested	Anesthesia introduction	CPB start	Pre tumor explant	Post tumor explant	CPB end	Normal range
Normetanephrine	145 pg/mL (791.7 pmol/L)	**435 230 pg/mL** **(2 376 355.8 pmol/L)**	**400 757 pg/mL** **(2 188 133.2 pmol/L)**	**153 271 pg/mL** **(836 859.7 pmol/L)**	**10 787 pg/mL** **(58 897.0 pmol/L)**	506.0 pg/mL (<2762.76 pmol/L)
Metanephrine	47 pg/mL (238.3 pmol/L)	**70 251 pg/mL** **(356 172.6 pmol/L)**	**78 560 pg/mL** **(398 299.2 pmol/L)**	**49 821 pg/mL** **(252 592.5 pmol/L)**	**1332 pg/mL** **(6753.2 pmol/L)**	< 130.0 pg/mL (< 659.1 pmol/L)

Abnormal values are shown in bold font. Values in parenthesis are International System of Units (SI).

Abbreviation: CPB, cardiopulmonary bypass.

In conclusion, we demonstrate the successful use of partial CPB and blood purification therapy (see Table 1) to stabilize intraoperative hemodynamics during the resection of a giant pheochromocytoma.

## Learning Points

- Pheochromocytoma should be included in the differential diagnosis of patients presenting with heart failure of unknown cause and associated hemodynamic instability.
- Preoperative administration of metyrosine may stabilize blood pressure in patients with giant pheochromocytomas who are susceptible to releasing large amounts of catecholamines, facilitating smoother transitions to invasive therapy.
- In surgical resection of giant pheochromocytomas in patients with a history of heart failure, the use of partial CPB and blood purification therapy with hemodiafiltration can help maintain stable hemodynamics and serve as valuable adjuncts.

## Data Availability

Original data generated and analyzed during this study are included in this published article.

## Abbreviations

CPB: cardiopulmonary bypass
PPGL: pheochromocytoma/paraganglioma
